# Drug cost avoidance analysis of cancer clinical trials in Spain: a study on cost contributors and their impact

**DOI:** 10.1186/s12913-022-08222-9

**Published:** 2022-07-26

**Authors:** Domingo Antonio Sánchez Martínez, Federico Salas-Lucia, Hanzi Jiang, Paula Ruiz-Carreño, José Luis Alonso Romero

**Affiliations:** 1 IMIB-Arrixaca. Medical Oncology Service, Hospital Clínico Universitario Virgen de La Arrixaca, Murcia, Spain; 2 Department of Medicine, The University of Chicago, Chicago, IL USA; 3 West Side United, Chicago, IL USA

**Keywords:** Hospital management, Linear regression, Healthcare costs, Sustainability, Clinical Trials, Oncology

## Abstract

**Objective:**

Analyze the cost contributors and their impact on the drug cost avoidance (DCA) resulting from cancer clinical trials over the period of 2015–2020 in a tertiary-level hospital in Spain (HCUVA).

**Methods:**

We performed a cross-sectional, observational, retrospective study of a total of 53 clinical trials with 363 patients enrolled. We calculated the DCA from the price of the best standard of care (i.e.: drugs that the institution would otherwise fund). A linear regression model was used to determine cost contributors and estimate their impact.

**Results:**

The total DCA was ~ 4.9 million euros (31 clinical trials; 177 enrollees), representing ~ 30% and ~ 0,05% approximately of the annual pharmaceutical expenditures at the HCUVA and for the Spanish Health System, respectively. Cancer type analysis showed that lung cancer had the highest average DCA by trial, indicating that treatments in these trials were the most expensive. Linear regression analysis showed that the number of patients in a trial did not significantly affect that trial's DCA. Instead, cancer type, phase trials, and intention of treatment were significant cost contributors to DCA. Compared to digestive cancer trials, breast and lung trials were significantly more expensive, (*p* < 0.05 and *p* < 0.1, respectively). Phase III trials were more expensive than Phase II (*p* < 0.01) and adjuvant trials were less expensive than palliative (*p* < 0.05).

**Conclusion:**

We studied cost contributors that significantly impacted the estimated DCA from cancer clinical trials. Our work provides the groundwork to explore DCA contributors with potential to enhance public relations material and serve as a negotiating tool for budgeting, thus playing an important role to inform decisions about resource allocation.

**Supplementary Information:**

The online version contains supplementary material available at 10.1186/s12913-022-08222-9.

## Introduction

Cancer has one of the most significant impacts on health budgets [1, 2], and estimations predict that the number of new cases will increase over the next two decades to 29.5 million per year by 2040 [3]. Worryingly, the lack of situation analysis and budgeting concerning this pathology has been identified as one of the main obstacles threatening the sustainability of health systems [4]. Recent studies propose clinical trials as an alternative new element to introduce sustainability into the Health System [5], aiming to prevent economic failure. Clinical trials investigate new diagnosing, treating, and managing cancer to improve the standard of care treatment [6]. For patients, the benefits of clinical trials include gaining access to experimental treatments when no other options exist and to new therapies not yet available [7], while contributing to the advancement of medical research. However, the economic benefits of running oncologic clinical trials for hospital administrators are often not considered. One potential financial benefit of clinical trials is drug cost avoidance (DCA) [8, 9], resulting when trial subjects receive industry-sponsored treatment drugs that the institution would otherwise fund [10]. Previous studies have quantified DCA derived from cancer clinical trials in different settings [11, 12], proven their efficacy to alleviate the economic pressure in the health system. However, there are no studies on cost contributors and their impact on DCA to the best of our knowledge. The purpose of this study was to analyze the cost contributors and their impact on the DCA resulting from cancer clinical trials throughout 2015–2020 in a tertiary-level hospital in Spain (HCUVA).

## Methods

### Identifying eligible trials

We reviewed clinical trials performed from 2015 to 2020 at the oncology department of HCUVA. Trials were excluded when (i) no patients were enrolled, (ii) screening failed, (iii) DCA could not be calculated due to insufficient drug information, and (iv) were observational phase 4 trials in which the experimental treatment was not industry-sponsored.

### Calculating DCA

Pharmacy and patient records for eligible trials were reviewed to determine each subject's treatment duration and quantity of the drug. Because the cost of the experimental drug in the clinical trials was unavailable, we calculated the best standard of care DCA using a reference price list. Depending on the treatment, the price was obtained from the Royal Decree of that year [13–18]. We used either the ex-factory, or the retail price depending upon the commercial status of the treatment. Additionally, some of the prices were provided by the hospital pharmacy service. The latter also provided the annual pharmaceutical expenditures at the Medical Oncology Department of the HCUVA. The drugs established in the standard of care concept for the DCA calculation were used for all the trials included in Table 1. The control non-innovative treatment was used for phase III trials as a reference. Because the clinical trials were blind, the number of cycles administered during the experimental and control treatment were the same. Thus, the number of cycles in Table 1 records the actual number administered. For phase II trials, the treatment was selected based on local clinical guidelines. When the standard of care drug dosage depends on the body surface area, we used standardized parameters for men and women (1.96 kg/m2 and 1.68 kg/m2, respectively) [19]. Equation [1] shows how we calculated the DCA.

**Table 1:** DCA per trial

Trial	Year	Cancer Type	Phase	Patients (N)	Female (%)	Cycle of Standard of Care	Price /Cycle (€)	Cycles (N)	DCA (€)
1	2015	Breast	III	6 | 2	100	Letrozol | Tamoxifen	59 | 2.35*	65 | 26	3,896.10
2	2015	Digestive	III	4	25	Paclitaxel	345*	28	9,660.00
3	2016	Gynecological	III	7	100	Carboplatin + Doxorubicin Liposomal	1045**	35	36,575.00
4	2016	Breast	III	3	100	Fulvestrant	176.4*	65	11,466.00
5	2016	Lung	III	6	50	Carboplatin-Pemetrexed + Pemetrexed	9.992 + 2.375**	37	72,894.00
6	2016	Lung	III	7	0	Nivolumab	7238**	203	1,469,314.00
7	2017	Breast	III	9 | 5	100	Letrozol | Tamoxifen	59 | 2.35*	341 | 161	20,497.35
8	2017	Breast	II	4	100	Fulvestrant	176.4*	31	5,468.40
9	2017	Breast	II	4	100	Capecitabine	43*	30	1,290.00
10	2017	Breast	III	8	100	Ribociclib + Letrozol	4772**	214	1,021,208.00
11	2018	Digestive	III	2	0	Folfiri + Bevacizumab	2896**	21	60,816.00
12	2018	Lung	III	1	0	Atezolizumab	4726**	16	75,616.00
13	2018	Sarcoma	II	5	20	Trabectedin	5820**	27	157,140.00
14	2018	Breast	II	1	100	Eribuline	1455**	15	21,825.00
15	2018	Gynecological	III	15	100	Carboplatin + Paclitaxel	338*	361	122,018.00
16	2018	Gynecological	III	17	100	Cisplatin + Paclitaxel	409*	182	74,438.00
17	2018	Breast	III	5	100	Paclitaxel + Pertuzumab + Trastuzumab | Letrozol	8640 | 4960 **	198	984,370.00
18	2019	Breast	III	9	100	Adriamycin + Cyclophosphamide + Paclitaxel | Pertuzumab + Trastuzumab	11384 | 4632**	148	466,832.00
19	2019	Digestive	III	5 | 2	14	FOLFOX | FOLFIRI	416 | 139*	96 | 30	44,106.00
20	2019	Digestive	III	5	0	FOLFOX	416*	29	12,064.00
21	2019	Gynecological	III	5	100	Olaparib	2646**	42	111,132.00
22	2019	Digestive	III	6	33	Gemcitabine + Nab-paclitaxel	449**	25	11,225.00
23	2019	Breast	III	10 | 6	100	Letrozol | Tamoxifen	92.1 | 2.35*	101 | 69	9,477.00
24	2019	Breast	III	7	100	Adriamycin + Cyclophosphamide + Paclitaxel	224**	96	21,504.00
25	2019	Melanoma	II	2	0	Pembrolizumab	3566**	17	60,622.00
26	2019	Prostate	III	2	0	Enzalutamide	3358**	14	47,012.00
27	2020	Lung	III	2	50	Carboplatin + Etoposide	71*	17	1,207.00
28	2020	Digestive	III	1	0	Lenvatinib	2249**	10	22,490.00
29	2020	Breast	II	1	100	Carboplatin + Gemcitabine	249*	4	996.00
30	2020	Gynecological	III	1	100	Topotecan	300*	3	900.00
31	2020	Breast	II	2	100	Adriamycin + Cyclophosphamide + Paclitaxel	3072**	2	6,144.00
								**Total**	**4,964,202**

*Ex-factory price, without the hospital discount (7.5 %). ** Price provided by the HCUVA Pharmacy servic

1 $$DCA=Price\;per\;cycle\;\times\;Number\;of\;cycles\;per\;trial$$

### Backward variable selection steps and Linear regression analysis

The original dataset contained seven trial characteristics: number of patients, number of cycles, price per cycle, cancer type, trial phases, intention of treatment and female percentage. The variables number of patients, price per cycle, number of cycles, and female percentage are numeric, and the other three variables are composed of characteristic values. We created indicator variables to translate characteristic values into numeric (e.g., indicator column for breast cancer will indicate breast cancer as 1, other cancer types as 0). Female percentage was not considered due to its high correlation with the breast cancer indicator variable, a multicollinearity situation that can mislead regression results. For similar reasons, we also excluded the price per cycle since due to its linear relationship with the number of cycles [1]. Next, we conducted a backward variable selection steps via linear regression to identify if any of the five remaining characteristics had no significant effect on the DCA. All remaining variables to consider have a *p* < 0.1 For the non-significant characteristics, changing their values, will likely not affect the outcome variable DCA. The backward variable selection steps allowed us to simplify the linear regression model.

In our linear regression model illustrated in Eq. [2], the significant characteristics (cancer type, trial phases, and intention of treatment), were selected as control variables. We set our baseline constant as digestive cancer, palliative treatment, and Phase II trials (β_0_; namely the intercept) to establish a comparison for the indicator variables. The dependent variable was the DCA from each trial. Using the 'lm' function of the R statistic software, the coefficient estimates (β) of each variable represents the magnitude of its impact on trial DCA while holding other factors constant.

2 $$DCA=\beta_0+\beta_1CancerType+\beta_2Intention+\beta_3Phase+\varepsilon_i$$

The variables were considered significant when *p* < 0.1, and to further interpret the results and its impact on DCA, we verified that the model fulfilled the following linear regression assumptions: (i) linear relationship between control variables and dependent variable, (ii) control variables are independent from each other, (iii) residual errors have a mean value of zero and (iv) residual errors have constant variance.

## Results

### Study sample

**Fig. 1:**
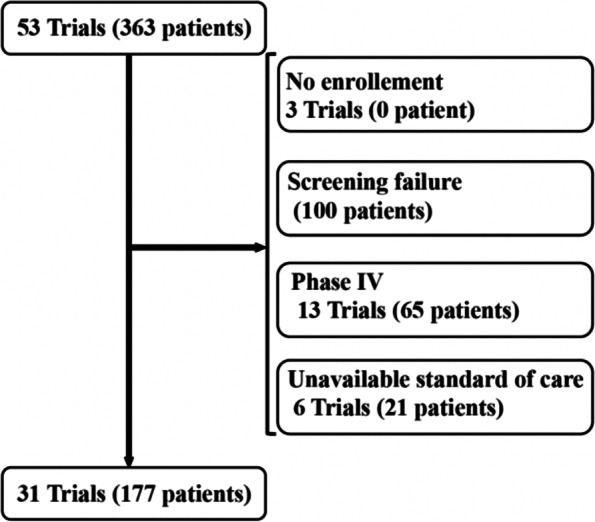
Flow chart of the process to identify eligible trials

We reviewed 53 industry-sponsored clinical trials with a total of 363 patients enrolled, from 2015 to 2020, at the oncology department of HUCVA. We excluded three trials that had no patient enrolled, 13 trials (65 patients) were in phase IV, for six trials (21 patients), we could not calculate the DCA and 100 patients failed the screening. The final sample studied included 31 trials, enrolling 177 patients (Fig. 1). The number of patients per study ranged from 1 to 17. Trials were distributed among seven tumor groups; the most common was breast cancer (13 trials), followed by digestive (6), gynecology (5), lung (4), prostate (1), melanoma (1), and sarcoma (1). The final sample had phase II and III trials (7 and 24, respectively) with palliative (25), adjuvant (4) and neoadjuvant (2) intentions.

### Drug cost avoidance

The DCA in all trials was calculated by the price of the standard treatment, including a total of 35 drugs. The estimated DCA during the study period was ~ 4.9 million euros (Table 1). The median estimated DCA per year was 827,367 € and 26,689 € per trial. DCA in this study represents approximately 30% and 0,05% of the annual pharmaceutical expenditures at the Medical Oncology Department at HCUVA and for the Spanish Health System [20], respectively. We found that DCA did not increase proportionally to the number of trials or number of patients (Table 2). For instance, in 2016, the DCA was 1,6 million euros from 4 trials and 23 patients, while in 2019, it was 0.78 million euros from 9 trials and 59 patients. Only 6.5% of DCA came from Phase II and 93.5% from phase III. Regarding the intention of clinical trials, palliative trials represented 89.2% of the DCA, followed by neoadjuvant and adjuvant (9.3% and 1.5%, respectively). Next, we conducted a cancer type analysis of the DCA and found that breast cancer had the highest DCA (Table 3). However, lung cancer had the highest average DCA by trial, and it was twice as much the breast cancer's, indicating that, on average, lung cancer treatments were the most expensive (Table 3). Digestive cancer had the least expensive treatments on average.

**Table 2:** Annual number of clinical trials, patients, and DCA

**Year**	**Trials (N)**	**Enrollments (N)**	**DCA(€)**
2015	2	12	13,556
2016	4	23	1,590,249
2017	4	30	1,048,463
2018	7	46	1,496,223
2019	9	59	783,974
2020	5	7	31,737

**Table 3:** Cancer type analysis of the DCA

Cancer Type	Trials (N)	Enrollments (N)	DCA(€)	Average DCA/trial (€)
Breast	13	82	2,574,973	198,074
Digestive	6	25	160,361	26,726
Gynecological	5	45	345,063	69,012
Lung	4	16	1,619,031	404,757
Melanoma	1	2	60,622	60,622
Prostate	1	2	47,012	47,012
Sarcoma	1	5	157,140	157,140

### Cost contributor's analysis

First, our backward variable selection steps via linear regression showed that the coefficient estimate for the number of patients and the number of cycles were insignificant (significance level at *p* < 0.1; *p*-values of 0.88 and 0.13, respectively; Supplementary Table 1), and did not affect trials’ DCA. These results allowed us to simplify the regression model in our further analysis. Next, we wished to investigate the impact magnitude of each significant cost contributor on the DCA based on the final regression model. We found a significant impact (*p* < 0.1) on cancer type, phase trials, and intention of treatment (Table 4). Specifically, compared to digestive cancer trials, breast and lung cancer trials were 619,264 € (*p* < 0.05) and 378,031 € (*p* < 0.1) more expensive, respectively. Sarcoma cancer trials were 749,242 € more expensive, respectively. Similarly, Phase III trials were 621,906 € (*p* < 0.01) more expensive than Phase II, and regarding the intention of the trials, adjuvant trials were 618,828 € (*p* < 0.01) less expensive than palliative (Table 4).

**Table 4:** Linear Regression Model Summary

Predictors	Estimates	DCA	
		*CI*	*p*
(Intercept)	-592102.01 **	-1121326.10 — -62877.91	0.030
Phase_III	618828.84 ***	166524.94 — 1071132.73	0.010
Breast	619264.36 **	164276.14 — 1074252.58	0.010
Lung	378030.92 *	-56420.82 — 812482.65	0.085
Gynecological	42285.77	-365266.09 — 449837.62	0.831
Melanoma	652724.01	-203473.45 — 1508921.46	0.128
Prostate	20285.17	-706691.63 — 747261.97	0.954
Sarcoma	749242.01 *	-106955.45 — 1605439.46	0.083
Adjuvant	-632147.69 **	-1126883.92 — -137411.46	0.015
Neoadjuvant	-100088.77	-640695.33 — 440517.78	0.704
**Observations**	31		
**R² / R² adjusted**	0.402 / 0.146		

* *p* < 0.1 ** *p* < 0.05 *** *p* < 0.01

## Discussion

This paper estimated the DCA from industry-sponsored oncological clinical trials that took place in HCUVA between 2015 and 2020. To estimate the DCA, we used the price of the best standard of care, resulting in ~ 5.1 million euros. Other DCA estimations performed in a single institution have shown similar saving percentages for the hospital or institution budget [11, 12]. Thus, supporting the idea that the DCA from oncological clinical trials provides economic savings that helps to sustain local health institutions in the short term.

A limitation of our and previous studies is that other non-pharmaceutical costs were not considered, implying the total savings derived from oncological clinical trials could be underestimated. In addition, DCA from experimental clinical trials may be underestimated when calculated based on the price of the control treatments. For example, in the present study, phase III clinical trials’ treatment administered to the control groups could have been different outside of the trial context. Notwithstanding, control treatments were always an appropriate option for the patients enrolled. Additionally, although our calculations considered control trial treatment variations, we could not adjust the experimental trial treatments due to the specific trial designs. On the other hand, we have not deducted the 7.5% mandatory hospital discount which contributed to overestimate the DCA.

Our study contained a unique sample of trials that had a diverse number of enrollees, cancer types, intentions of the treatments and phase, making our DCA estimations difficult to be directly compared with other estimated DCA. For instance, previous studies have estimated DCA by variables such as the number of patients, the length, or phases of the trials [11], the pharmacological categories [14, 21, 22] or pathology type [22].

Our initial data exploration showed that variations in the number of clinical trials and patients enrolled per year did not represent a proportional variation in the annual DCA, incentivizing us to further investigate other potential DCA contributing factors. Our cancer type analysis of the DCA showed that lung cancer trials had the highest average DCA per trial compared with other cancer types, suggesting lung cancer treatments could be the most expensive. This agrees with the observation that the DCA per patient varies drastically among tumor groups [23]. We suspected that other trial characteristics, such as treatment intention and phases, could also have significant impacts on DCA. Therefore, to further investigate and isolate each individual characteristic’s impact on DCA, we constructed a linear regression model as illustrated in Eq. [2]. The sensitivity analysis shows that the number of cycles and the number of patients enrolled in a trial had a non-significant contribution to DCA, which agrees with our previous observation. Thus, suggesting other contributors could carry more weight on influencing DCA. Indeed, our linear regression analysis confirmed that certain cancer types, trial phases, and treatment intention had a significant impact on DCA. Specifically, we found that palliative trials tend to be 633,334 € (*p* < 0.05) more expensive than adjuvant trials while holding other variables constant. Similarly, breast and lung cancer trials were 624,940 € (*p* < 0.001) and 378,031 € (*p* < 0.1) more expensive than digestive cancer trials, respectively. Although we found that Melanoma and Sarcoma cancer trials have significant impact towards DCA, one should exercise caution when interpreting the practical significance of these results as our dataset only included one trial per each of these cancer types. These findings could be explained by the varying dosage requirements for differing trials, the length of time patients remain on trial drugs due to progression of disease, and the cost of the treatments. For example, in our study, each treatment cycle’s price between trials ranged from 43 € to 8640 €. These price differences reflect that innovative therapies, such as cyclin inhibitors and immunotherapy, are significantly more costly than other standard chemotherapies [11, 24]. The increasing number of new options for prevention, diagnosis, and treatment, makes studies of the DCA of the new therapies increasingly important to inform decisions about resource allocation. Our study might be useful for hospital management by providing a projection on future DCA derived from clinical trials based on their characteristics.

## Conclusion

We studied cancer clinical trial characteristics and found significant cost contributors that impacted the estimated DCA. Our work provides the groundwork to explore DCA contributors with the potential to enhance public relations material and serve as a negotiating tool for budgeting, thus playing an essential role in informing resource allocation decisions.

## Supplementary Information

**Additional file 1: Supplementary Table 1.** Sensitivity Analysis

## Data Availability

All data generated or analyzed during this study are included in this published article.
